# Whole-Genome Sequencing Identifies an Atypical *Listeria monocytogenes* Strain Isolated from Pet Foods

**DOI:** 10.1128/genomeA.01243-14

**Published:** 2014-12-24

**Authors:** Laurel S. Burall, Christopher Grim, Gopal Gopinath, Pongpan Laksanalamai, Atin R. Datta

**Affiliations:** Center for Food Safety and Applied Nutrition, Food and Drug Administration, Laurel, Maryland, USA

## Abstract

Four *Listeria* isolates, including an atypical strain, were isolated from various pet foods and sequenced. We report here the draft genome sequences of these isolates and a comparative genomic analysis with a closely related human clinical isolate. An analysis of the atypical strain identified a frameshift mutation in the *prfA* gene.

## GENOME ANNOUNCEMENT

*Listeria monocytogenes* is a Gram-positive bacterium that is the causative agent of listeriosis, a disease with a high mortality rate, caused by the consumption of contaminated foods. The development of new pet foods, particularly raw foods, has raised concerns that these foods might contain microbial pathogens and pose a health risk to pets and their owners, leading to a surveillance effort that isolated several *Listeria* strains (1). One of these pet food isolates (LS885 [12FM-201]), although lacking hemolysin on blood agar, produced an *hly*-specific amplicon by a PCR method (2). We further confirmed that LS885 also lacked phospholipase C on RAPID’L.mono (Bio-Rad, Hercules, CA), but it was identified as *L. monocytogenes* by API *Listeria* tests (bioMérieux, Durham, NC). Whole-genome sequencing was undertaken of this isolate as well as for three other pet food isolates: two *L. monocytogenes* isolates (LS884 [12-FM-196] and LS889 [12-FM-215]) and an *Listeria innocua* isolate (LS888 [12-FM-212]). All three of the *L. monocytogenes* pet food isolates were characterized as serotype 1/2a. A pangenomic microarray comparison (data not shown) identified a close relationship between LS885 and LS743, a serotype 1/2a isolate from the 2011 cantaloupe outbreak (3), which led us to sequence and compare this isolate with LS885.

Genomic DNA was extracted using the DNeasy blood and tissue kit (Qiagen, Valencia, CA), as described previously (4). Libraries were prepared using a Nextera XT sample preparation kit (Illumina, San Diego, CA), and a 2 × 250 paired-end sequencing run was performed on an Illumina MiSeq. The reads were trimmed and assembled using the CLC Genomics Workbench version 7.0 (CLC bio, Germantown, MD). The genome sizes averaged around 2.9 Mb, with a G+C content of 37 to 38%. The assemblies have >30× coverage and contain 17, 15, 12, 17, and 9 contigs, representing the genomes of LS884, LS885, LS888, LS889, and LS743, respectively. The contigs were annotated using the Rapid Annotations using Subsystems Technology (RAST) server (5, 6), which identified 2,914, 2,935, 2,870, 3,001, and 2,832 coding sequences for LS884, LS885, LS888, LS889, and LS743, respectively. As naturally occurring *prfA* mutations have been linked to a loss of virulence phenotypes (7, 8), the *prfA* gene was identified in all five strains, and the sequences were aligned using Clustal X2 (9). The alignment identified a single base pair deletion in the LS885 *prfA* gene, leading to a truncated protein. A comparison of LS885 with LS743 indicated that the vast majority of the genes are very similar. However, in addition to a few isolated variations, two sections of phage sequence containing 96 open reading frames (ORFs) were present in LS885 only. Based on their contig positions, it is possible that these two regions represent one phage insertion. These data add to the understanding of the genetic profile of *L. monocytogenes* from different pet foods and also establish a close sequence homology with a pet food and a human clinical isolate.

### Nucleotide sequence accession numbers.

This whole-genome shotgun project has been deposited in DDBJ/EMBL/GenBank under the accession numbers JRYY00000000, JRYZ00000000, JRYX00000000, JRZA00000000, and JRZB00000000 for *Listeria* strains LS884, LS885, LS888, LS889, and LS743, respectively.
